# Selection signatures of wool color in Gangba sheep revealed by genome-wide SNP discovery

**DOI:** 10.1186/s12864-024-10464-2

**Published:** 2024-06-17

**Authors:** Wentao Zhang, Cuicheng Luosang, Chao Yuan, Tingting Guo, Caihong Wei, Jianbin Liu, Zengkui Lu

**Affiliations:** 1 Key Laboratory of Animal Genetics and Breeding On Tibetan Plateau, Ministry of Agriculture and Rural Affairs, Lanzhou Institute of Husbandry and Pharmaceutical Sciences, Chinese Academy of Agricultural Sciences, Lanzhou, 730050 China; 2 Sheep Breeding Engineering Technology Research Center of Chinese Academy of Agricultural Sciences, Lanzhou, 730050 China; 3 State Key Laboratory of Animal Biotech Breeding, Institute of Animal Sciences, Chinese Academy of Agricultural Sciences, Beijing, 100193 China; 4 Institute of Animal Science, Tibet Academy of Agricultural and Animal Husbandry Sciences, Lhasa, 850009 China

**Keywords:** Gangba sheep, Tibetan sheep, Wool color, Whole genome resequencing, Selection signature

## Abstract

**Background:**

Gangba sheep as a famous breed of Tibetan sheep, its wool color is mainly white and black. Gangba wool is economically important as a high-quality raw material for Tibetan blankets and Tibetan serge. However, relatively few studies have been conducted on the wool color of Tibetan sheep.

**Results:**

To fill this research gap, this study conducted an in-depth analysis of two populations of Gangba sheep (black and white wool color) using whole genome resequencing to identify genetic variation associated with wool color. Utilizing PCA, Genetic Admixture, and N-J Tree analyses, the present study revealed a consistent genetic relationship and structure between black and white wool colored Gangba sheep populations, which is consistent with their breed history. Analysis of selection signatures using multiple methods (F_ST_, π ratio, Tajima's D), 370 candidate genes were screened in the black wool group (GBB vs GBW); among them, *MC1R, MLPH, SPIRE2, RAB17, SMARCA4, IRF4, CAV1, USP7, TP53, MYO6, MITF, MC2R, TET2, NF1, JAK1, GABRR1* genes are mainly associated with melanin synthesis, melanin delivery, and distribution. The enrichment results of the candidate genes identified 35 GO entries and 19 KEGG pathways associated with the formation of the black phenotype. 311 candidate genes were screened in the white wool group (GBW vs GBB); among them, *REST, POU2F1, ADCY10, CCNB1, EP300, BRD4, GLI3*, and *SDHA* genes were mainly associated with interfering with the differentiation of neural crest cells into melanocytes, affecting the proliferation of melanocytes, and inhibiting melanin synthesis. 31 GO entries and 22 KEGG pathways were associated with the formation of the white phenotype.

**Conclusions:**

This study provides important information for understanding the genetic mechanism of wool color in Gangba, and provides genetic knowledge for improving and optimizing the wool color of Tibetan sheep. Genetic improvement and selective breeding to produce wool of specific colors can meet the demand for a diversity of wool products in the Tibetan wool textile market.

**Supplementary Information:**

The online version contains supplementary material available at 10.1186/s12864-024-10464-2.

## Background

The Gangba sheep grows on the Qinghai-Tibet Plateau, distributed at an altitude of 4,300 m to 6,800 m above sea level, which is a unique livestock genetic resource in China. Gangba sheep belong to the transition type between river valley type Tibetan sheep and plateau type Tibetan sheep, which is one of the excellent Tibetan sheep known in Tibet. Gangba wool has long wool fiber, strong weaving, high elasticity and tensile strength, glossy, large and thick set of hairs, less dry dead hairs, and is one of the finest qualities in the coarse wool type. Its coarse wool can be used to make one of the world's three most famous carpets, the Tibetan carpet (also known as khaki) [1–3]; its fluff and dual-type wool can be used to make the popular Tibetan serge and other hand-woven fabrics. Gangba wool color is mainly white, black, and brown, of which white is more, accounting for 69.5%, and brown is very little, accounting for only 1.5%.

With the development of technologies [4–8] such as sequencing and gene editing, more and more genes and variants related to hair color have been mined and validated. The formation of wool color involves several processes, including the development of pigment cells, pigment synthesis, and pigment transport and distribution. The *ASIP* [9], *TYR* [9–11], *TYRP1* [9, 12], *KIT* [13], *KITLG* [14, 15], and *PMEL* [16, 17] genes play crucial roles in wool color formation. Variants in these genes can influence melanin production, melanogenesis, cell differentiation, and migration, ultimately impacting the depth, hue, and patterns of wool color. Among Gangba wool products, white wool is highly favored by Tibetans due to cultural beliefs and ease of dyeing [18–20]. Therefore, breeding black wool into white color meets the market demand and has important economic benefits [20–23]. Wool color is a complex trait regulated by multiple genes [9, 18], and similar appearance may be controlled by different genetic mechanisms [9, 13, 24].

Considering the special historical origin and living environment of Tibetan sheep, the genes controlling wool color may differ from previous studies. Given the relative blank of wool color research in Tibetan sheep, we obtained comprehensive genetic data by whole genome resequencing of black and white Gangba sheep. Then, we used multiple signal analysis methods for joint screening to identify selection signals associated with the formation of black and white wool color on a genome-wide scale, and to identify the candidate genes and genetic variants that affect wool color. This advances our understanding of wool color genetic variation in Tibetan sheep at the genomic level and informs future breeding strategies.

## Materials and methods

### Sample collection and resequencing

To ensure a representative and comparable population, we selected adult healthy rams with pure black (or pure white) wool color and no stray spots. We select half-sibling individuals as samples to minimize the differences arising between individuals. These sheep come from the black sheep breeding farm in Kongma Township, Gamba County.

We collected blood from the jugular vein of black Gangba and white Gangba sheep (Table 1). DNA was extracted using the TIANamp Blood DNA Kit (Tian Gen Biotech Co. Ltd., Beijing, China). Genomic DNA purity and concentration were measured using a Nanodrop 2000 Nucleic Acid Protein Analyzer (Thermo, Scientific, Wilmington, NC, USA). Genomic DNA is repaired with flat ends after randomly interrupting it into short DNA fragments with enzymes. Then dA tails were attached to both ends of the DNA fragments and sequencing junctions were attached. The DNA fragments with junctions were purified by AMPure XP magnetic beads, and fragments in the range of 300–400 bp were selected for PCR amplification. The constructed library was purified, library checked, and sequenced on Hiseq X10 PE150. The resulting Raw reads were stored in FASTQ file format for subsequent analysis.

**Table 1:** Information of the sheep populations in this study

Sample	Abbreviation	Photo	Color	Sex	Age	Size
Black GangBa sheep	GBB		Black	Male	24 month old	20
White GangBa sheep	GBW		White	Male	24 month old	20

### Quality control and alignment

The steps of filtering [25] are as follows: (1) remove the reads containing adapter and retain the remaining reads; (2) remove the reads containing N ratio more than 10%; (3) remove the low-quality reads (the number of bases with quality value Q ≤ 20 accounted for more than 50% of the whole read). The reference genomes chosen for the comparison were Self-assembled genome_HB, which was used as the reference for all naming and annotation results. We used the MEN algorithm of the comparison software BWA (0.7.15) [26] to compare the high-quality sequencing data with the two reference genomes (parameters: -k 32 -M). The results of the comparison in SAM format were exported to a BAM format file via SAMtool. Next, after duplicate reads were labeled by Picard (2.18.7) software (http://sourceforge.net/projects/picard/), we used bedtools (v2.25.0) [27] software to count the coverage. We used ANNOVAR [28] for the functional annotation of the Variant. To improve the accuracy of data analysis, we filtered the raw SNPs and used the filtered high-quality SNP markers for selection signal analysis. The filtering conditions [29] were: the removal of marker loci with more than 20% missing rate and minor allele frequency (MAF) not less than 5%.

### Genetic variation

**Fig. 1:**
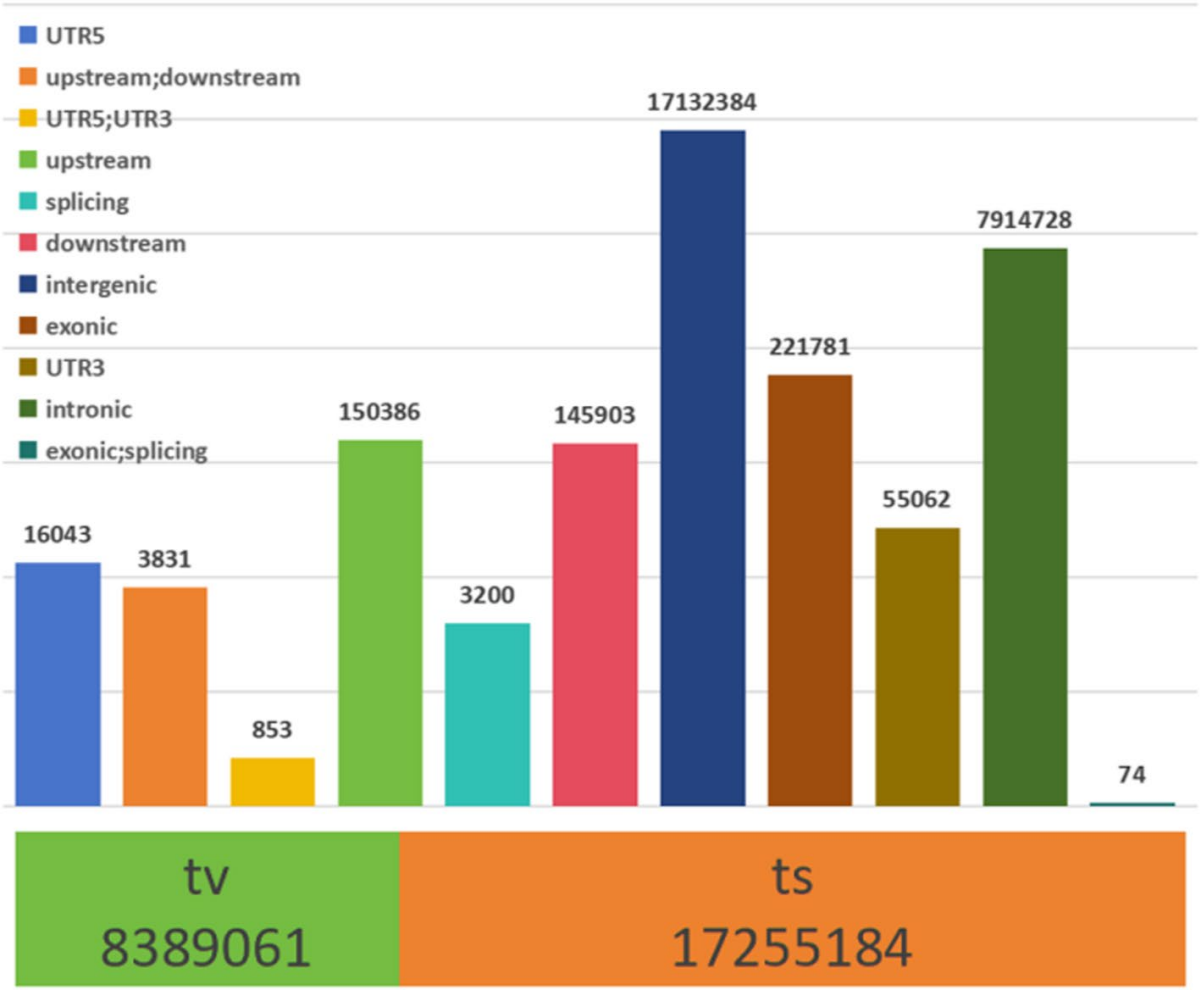
The distribution of SNP variants in the genome region. The upper bar graph represents location information; The lower stacked graph represents conversion and inversion information

In this study, 40 Gangba sheep were subjected to whole genome resequencing, and the average High-quality clean data of each sample after quality control was 15,477,184Kb (107,384,722.8 High-quality clean reads) (see Table S1; Table S2). The average total and perfect mapped ratio of Gangba sheep were 99.37% and 85.40%, respectively, and the average sequencing depth was 6.0 × (see Table S3; Table S4). After Variant annotation, a total of 25,644,245 SNPs were obtained (see Fig. 1). The TS/TV ratio was determined to be 2.06, indicating a standardized genomic population structure. After filtering the snp, a total of 2,507,942 high-quality SNPs were identified in 40 Gangba sheep, with 2,376,542 and 2,397,114 distributed in the GBB and GBW populations, respectively. Among them, a high percentage of 2,265,714 SNPs were common to both populations. These findings provide a reliable database for studying the population structure and identifying selection signals for potential wool color in Gangba sheep.

### Population structure analysis

In order to correct for the impact of LD, the filtered SNPs were trimmed using the indep-pairwise [30] function of the PLINK 1.09 software [31] (parameters: non-overlapping window of 25 SNPs, step size of 5 SNPs, and a threshold of 0.05 for r^2^). Principal component analysis (PCA) was performed by PLINK 1.09 to identify potential genetic clusters based on major variants. In addition, to infer the proximity of kinship and evolutionary history among populations, the neighbor-joining tree (N-J tree) was constructed by the neighbor-joining methods [32] of the treebest [33] software. Then, we used the ITOL software [34] (https://itol.embl.de/upload.cgi, accessed on 15 December 2023) to visualize it. In addition, in order to understand the evolutionary process, plink and frappe [35] software was used to construct the population genetic structure [29], with k values set from 2 to 4, to infer the population structure and individual genetic components.

### Selection signal analyses

In this study, three methods were used for selection elimination, including Pairwise Fixation Index (F_ST_) [36], π ratio [37, 38], and Tajima's D [39]. The combined F_ST_ and π ratio analysis can provide more integrated and comprehensive information on genetic dynamics, which can help to reveal possible selection signals and evolutionary patterns among and within different wool color populations. Then, Tajima's D was used to perform a neutral test analysis to find out whether genetic differentiation had occurred. Based on the filtered SNPs, intra-population F_ST_, Pi ratio and Tajima's D analyses were performed using PopGenome software [40, 41] with sliding windows by physical length in 100 kb windows and 10 kb steps [25].

### Detection and annotation of candidate genes

Next, we screened candidate selection signals for both wool colors. The highest 5% of F_ST_ and π ratio was used as a threshold to filter overlapping SNP loci. These overlapping loci are the candidate selection signal loci for the two wool colors. In addition, the lowest 5% of SNP loci analyzed by Tajima's D were used as candidate selection signals for both wool colors. A region of 50 kb upstream and downstream of these candidate sites was considered as the selection signal region [25]. ANNOVAR software (https://annovar.openbioinformatics.org/en/latest/, accessed on 10 December 2023) [28] was used to annotate the genes and the reference genome was sheep. All of the associated graphs have been drawn using R scripts [42]. Next, a Venn diagram [43] was constructed based on the candidate genes screened by F_ST_, π ratio and Tajima's D. Finally, the candidate genes for black and white wool color were obtained as shared genes by Venn diagram. Candidate genes within the sweep regions were extracted for further analysis.

### Candidate gene enrichment analysis

Performing Gene Ontology (GO) and Kyoto Encyclopedia of Genes and Genomes (KEGG) enrichment analyses helps to explain the regulatory mechanisms of genes in the expression of features such as wool color and provides key clues for subsequent studies. The candidate genes were functionally categorized by DAVID 6.8 [44] (https://david.ncifcrf.gov/, accessed on 7 January 2024), including molecular functions, biological processes, and cellular components. *Ovies aries* was chosen as the background for statistical analysis by the Hypergeometric test, with *p* < 0.05 as the significance threshold. Kobas 3.0 [45] (http://kobas.cbi.pku.edu.cn/kobas3/genelist/, accessed on 7 January 2024) was then used for pathway enrichment. The significance of the enriched pathways was assessed by the Hypergeometric test/Fisher's exact test with the *p*-value set at 0.05 in the context of *Ovies aries*. Next, we used Bioinformatics [46] (https://www.bioinformatics.com.co.uk, accessed on 10 January 2024) to produce GO-enriched bubble plots as well as Kegg-enriched gene-pathway Sankey plots. Finally, STRING (https://cn.string-db.org/, accessed on 17 January 2024) was used for gene interaction analysis of shared genes.

## Results

### Population genetic analysis

**Fig. 2:**
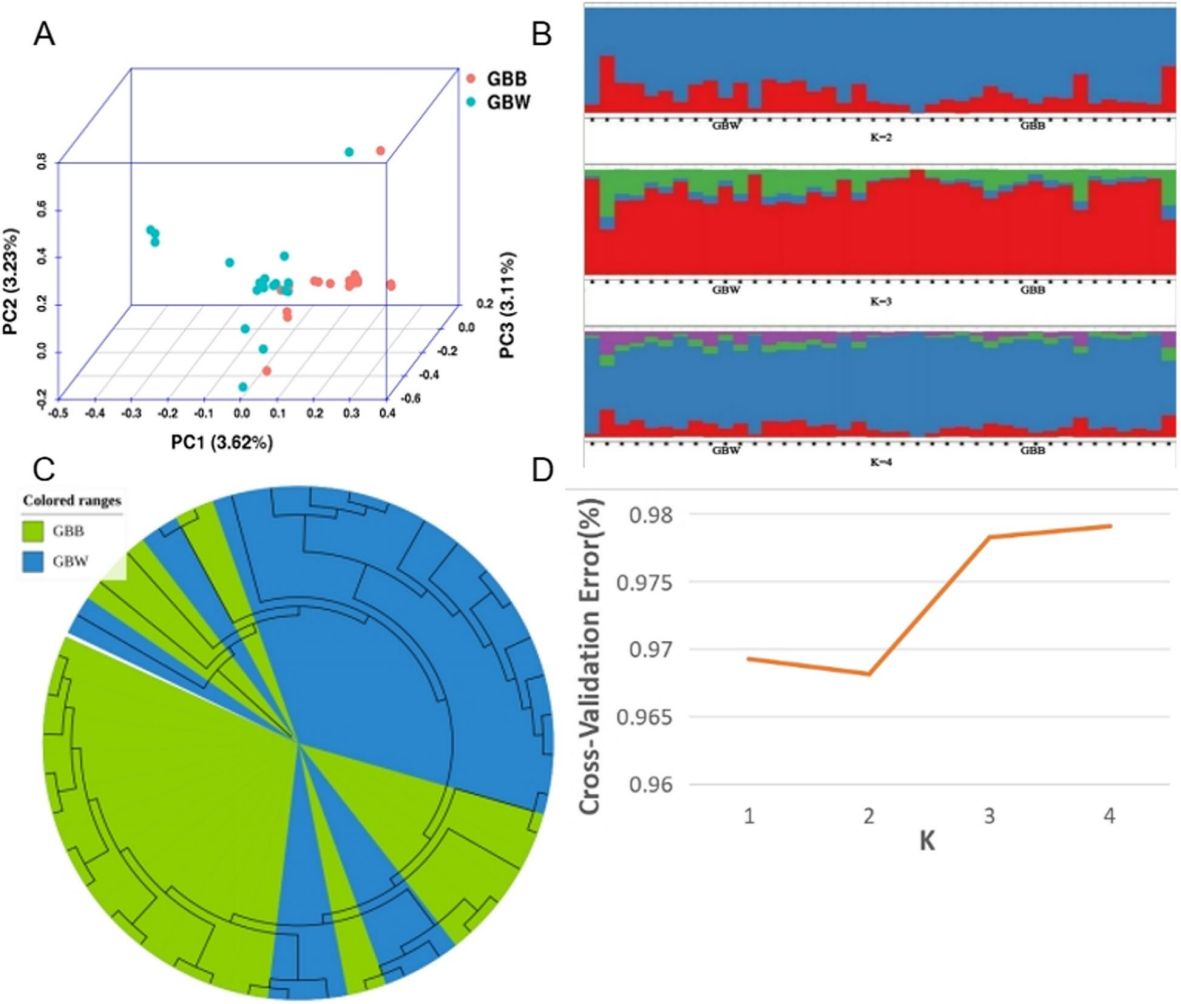
Analysis of the genetic structure of the population. **A** Principal component analysis (PCA); **B** K = 2 ~ 4, population structure analysis; **C** Individual evolutionary tree (N-J tree); **D** Cross-validation error

First, the proportion of variation explained by PC1, PC2, and PC3 is low, and there is some crossover between the two groups. Therefore, these two groups are the same population. Then, we can find that the two populations are not clearly distinguished from each other by the population genetic structure diagram (see Fig. 2B, D) at k = 2. GBW has slightly more genetic ancestry assigned to the "red background" compared to the GBB population. At k = 3, k = 4, the two populations are still not separated from the whole. The background composition of some of these individuals is more similar to that of the other population. The genetic admixture results are consistent with the PCA results, suggesting that the position of an individual in the genetic space and its genetic composition are consistent across genetic populations. Finally, the two populations were also found to be somewhat separated based on the N-J tree (see Fig. 2C) results, corroborating the PCA and genetic admixture results. We observed crossovers in the distance calculated (GBB8, GBB14, GBB20, GBW1, GBW7, GBW12, etc.), which is consistent with the crossover individuals in the previous PCA and the anomalous backgrounds of the constituent individuals in genetic admixture. This is yet another aspect that demonstrates the reliability of the population genetic analysis.

### Analysis of selection signals

**Fig. 3:**
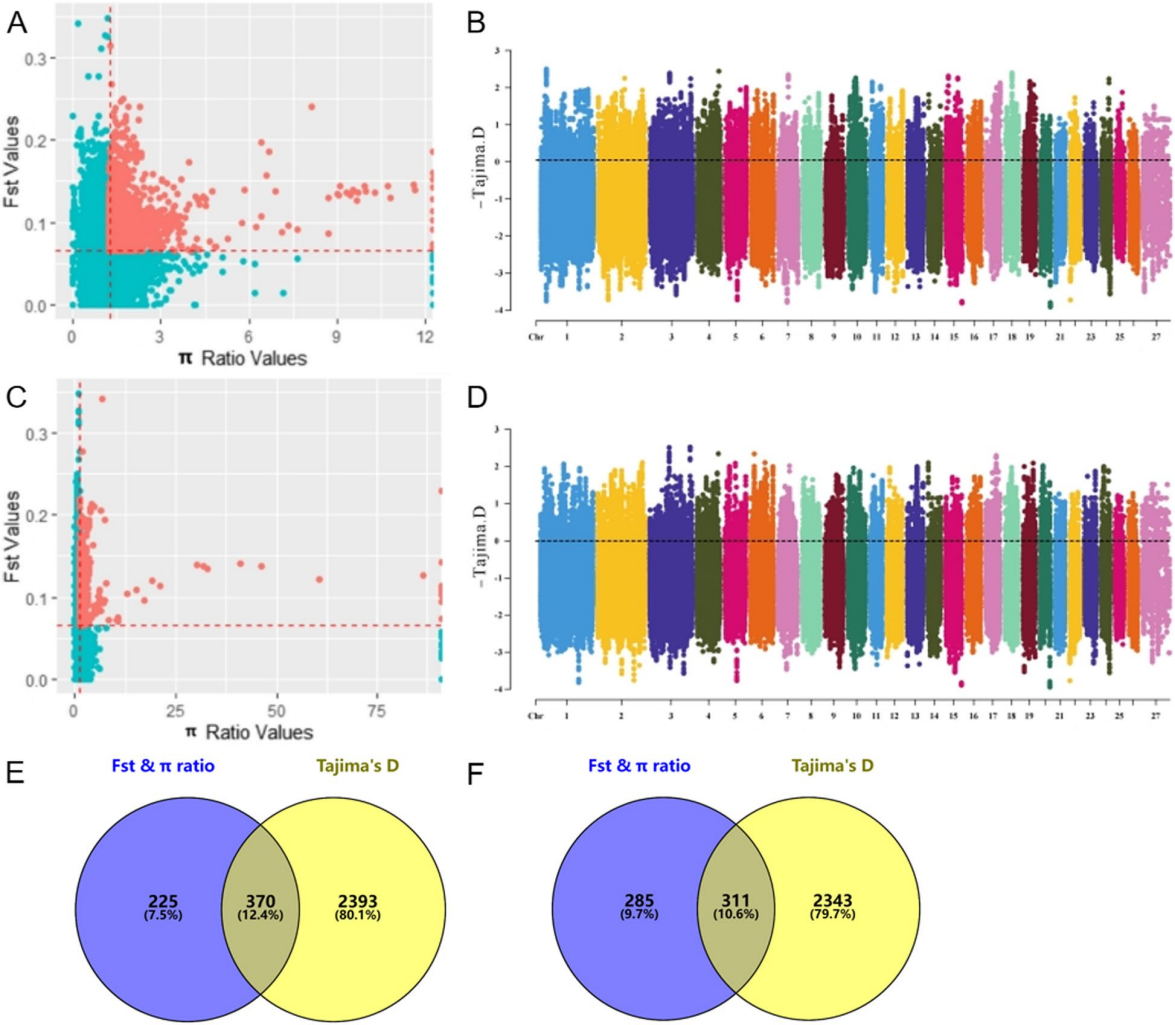
Selection signal analysis. **A** F_ST_ and π ratio joint selection elimination (GBB vs GBW); **B** Genome wide distribution of Tajima's D (GBB vs GBW); **C** F_ST_ and π ratio joint selection elimination (GBW vs GBB); **D** Genome wide distribution of Tajima's D (GBW vs GBB); **E** F_ST_ & π ratio and Tajima's D screened for overlapping genes (GBB vs GBW); **F** F_ST_ & π ratio and Tajima's D screened for overlapping genes (GBW vs GBB)

In the GBB vs GBW group, we jointly screened 2443 top 5% SNP loci by combined F_ST_ and π ratio analysis (see Fig. 3A). 12,915 SNP loci were screened by Tajima's D method (see Fig. 3B). These loci were used as potential selection signals by ANNOVAR for annotation. The two selection signal analysis methods identified 595 and 2763 candidate genes, respectively. Based on the Venn diagram, 370 overlapping candidate genes associated with black wool formation were screened (see Fig. 3E; Table S5), including *MC1R, MLPH, SPIRE2, RAB17, SMARCA4, IRF4, CAV1, USP7, TP53, MYO6, MITF, MC2R, TET2, NF1, JAK1, GABRR1,* etc. Similarly, in the GBW vs GBB group, 2718 and 12,973 potential selection signals were obtained by the combined F_ST_ and π ratio analysis (see Fig. 3C) and Tajima's D method (see Fig. 3D), respectively. After annotation, 596 and 2654 candidate genes were obtained, respectively. Finally, 311 overlapping candidate genes related to white wool formation were screened out using a Venn diagram (see Fig. 3F; Table S5), including *REST, POU2F1, ADCY10, CCNB1, EP300, BRD4, GLI3*, and *SDHA*, etc.

### Enrichment analysis

**Fig. 4:**
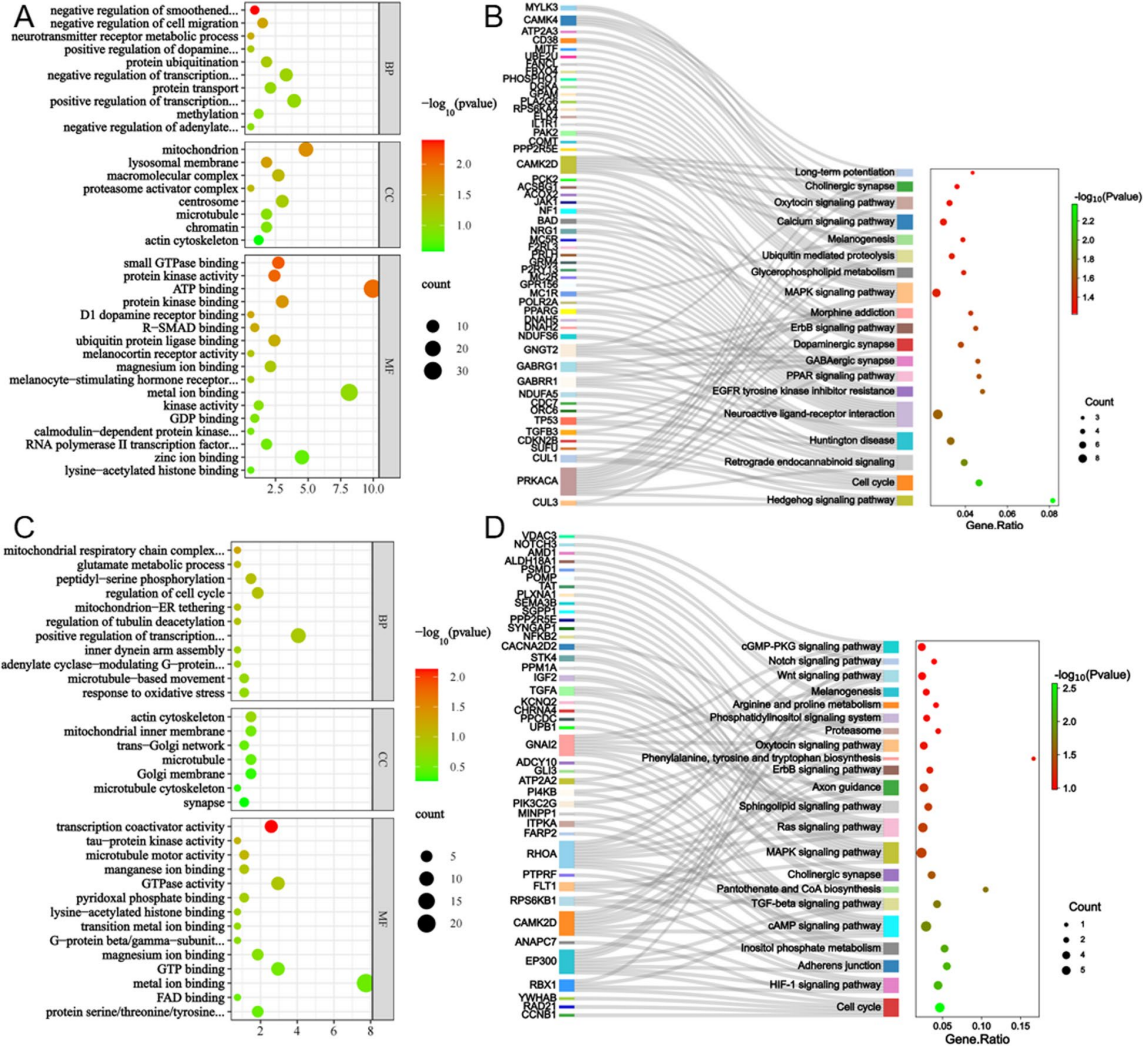
GO enrichment and KEGG enrichment results. **A** The top 30 GO terms (GBB vs GBW); **B** Sankey diagrams for KEGG pathway enrichment (GBB vs GBW); **C** The top 30 GO terms (GBW vs GBB); **D** Sankey diagrams for KEGG pathway enrichment (GBW vs GBB)

In the GBB vs GBW group (see Table S6; Table S7; Fig. 4A; B), the candidate genes were significantly enriched in 11 significant Biological Processes, 9 Cellular Components, and 13 Molecular Functions. 45 significant pathways were obtained by KEGG enrichment analysis. Based on GO terms (http://geneontology.org/, accessed on 10 January 2024), KEGG (https://www.kegg.jp/kegg/pathway.html, accessed on 10 January 2024), and previous studies, the following 35 GO terms and 19 Pathways were found to be associated with black wool formation: negative regulation of smoothened signaling pathway involved in dorsal/ventral neural tube patterning, proteasome activator complex, protein kinase activity, Hedgehog signaling pathway, EGFR tyrosine kinase inhibitor resistance, Melanogenesis, etc.

Similarly, in the GBW vs GBB group (see Table S8; Table S9; Fig. 4C; D), GO enrichment analysis yielded a total of 2 significant Biological Processes, 2 significant Cellular Components, and 1 significant Molecular Functions. 24 significant Pathways were obtained by KEGG enrichment analysis. A search revealed 31 GO terms (including: regulation of tubulin deacetylation, actin cytoskeleton, microtubule motor activity, etc.) and 22 Pathways (including HIF-1 signaling pathway, MAPK signaling pathway, Ras signaling pathway, etc.) are associated with white wool formation.

### Shared gene

**Fig. 5:**
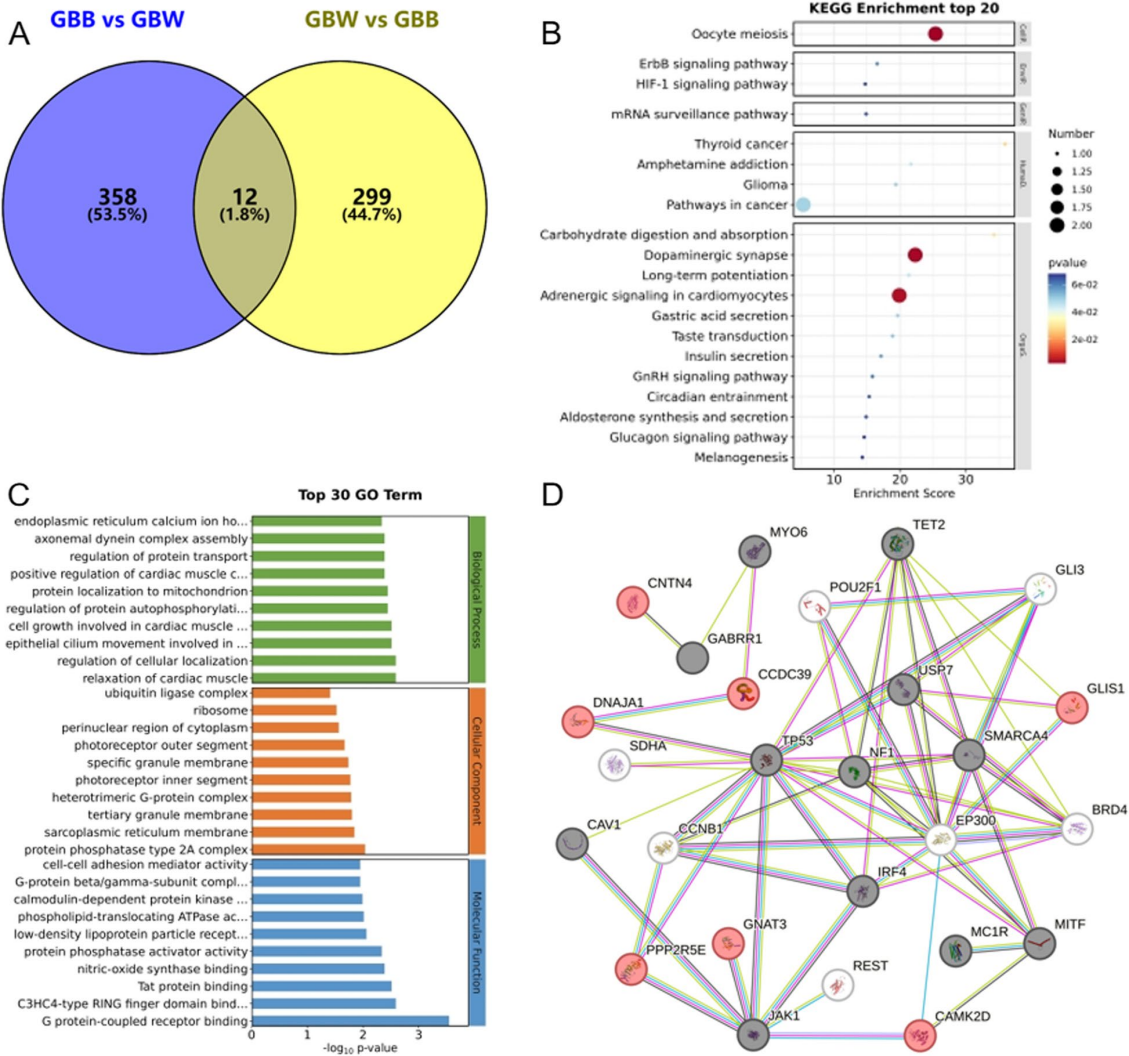
Enrichment analysis and interaction network of shared genes. **A** Venn diagram of shared genes; **B** KEGG pathway enrichment results; **C** GO function enrichment results; **D** Gene Interaction Network (red are shared genes, black and white are candidate genes for partial black and white wool, respectively)

We screened candidate genes associated with both wool colors by Venn diagram (see Fig. 5A), and obtained 12 overlapping shared genes (including: *GNAT3, UGT2C1, OLFR12, DNAJA1, RPS24, PPP2R5E, CNTN4, CCDC39, TFG, ATP8B4, GLIS1, CAMK2D*). Such genes, as genes associated with both black and white phenotypes, imply that these genes exhibit crossover effects in the phenotype of the organism. The results of GO and KEGG enrichment (see Fig. 5B; C; Table S10) analysis revealed that phospholipid transport (GO:0015914), Golgi apparatus (GO:0005794), GTPase activity (GO:0003924), Dopaminergic synapse (oas04728), Melanogenesis (oas04916), Wnt signaling pathway (oas04310) and so on are involved in signal transduction, melanin synthesis, and melanin granule transport. Selected black and white wool color candidate genes were analyzed for gene interactions with shared genes. It is found that the shared genes (*PPP2R5E, GLIS1, CAMK2D*) have gene function interactions with the two wool color candidate genes in the interaction network (see Fig. 5D).

## Discussion

Neutral theory and hitchhiking theory are the theoretical bases of selection signature. In recent years, with the development of high-throughput sequencing technology, researchers have mined a large number of functional genes related to economic traits by analyzing selection signals in livestock, providing more genetic information for molecular breeding of livestock [47–54].

### Genetic variation and population genetic analysis

The 99.37% high matching rate in sequencing indicates comprehensive coverage of the reference genome, ensuring accurate genomic analysis. An 85.40% higher perfect match rate reduces errors in variant detection. A sequencing depth of 6.0 × enhances confidence in variant annotation, mutation site identification, and genomic structure exploration. This study offers reliable data for Gangba sheep genomic analysis, supporting genetic and population studies. Shared SNPs suggest common genetic traits, with a high proportion indicating a shared ancestor. The low percentage of unshared SNPs improves the accuracy of subsequent wool color selection signal screens. A TS/TV ratio near 2 suggests a balanced genome with conserved structure, supporting reliable data for population structure and selection signal studies.

The PCA and N-J trees revealed a cross-mixing of the two populations. The difference in cross-validation error between K = 2 and K = 1 is tiny. And there is no significant difference in the composition of the two populations in genetic admixture. Considering the results together, we hypothesize that the GBB and GBW populations may share a common ancestor, consistent with the history and geography of the Gangba sheep.

### Analysis of selection signals related to wool color

The heritability of wool color is low, and genes associated with wool color may be located in regions of low genetic diversity [55–59]. To avoid neglecting candidate genes associated with wool color, we set the threshold for selection signal analysis to the top 5%. The formation of wool color is a complex process that includes pigment cell development [60, 61], pigment synthesis [60, 61], and pigment transport and particle distribution [9, 60–63].

#### Genes associated with black wool formation

Upon investigation, most of the genes in the black wool group are associated with melanocyte formation (*MITF, GABRR1, SMARCA4*), melanin synthesis (*MC1R, SMARCA4, IRF4, CAV1, USP7, TP53, MITF, MC2R, TET2, NF1, JAK1*), melanin transport (*MLPH, SPIRE2, MYO6, RAB17*). *MITF* not only directly controls *DCT, PMEL17, TYRP1,* and *TYR* to regulate pigmentation [64], but also regulates the expression of genes related to pigmentation in some indirect ways (e.g., the Melanogenesis pathway). The *SMARCA4* gene is a co-activator of the *MITF* genes [65], which influences the formation of melanocytes as well as pigmentation synthesis. This was also verified by the STRING gene interaction map. Overexpression of *GABRR1* [66] inhibits melanin stem cell regeneration through multiple signal transduction pathways. The *MC1R* gene is a gene that encodes a melanin-stimulated activated receptor, which plays a key role in the production and distribution of melanin. In contrast, *MC2R*, as the only isoform of the MCR that binds only to adrenocorticotropic hormone (ACTH) but not melanocyte-stimulating hormone (MSH) [67], affects melanin production. *IRF4* affects melanin deposition by regulating tyrosinase expression and interferon-γ responses [68]. *CAV1* deficiency specifically affects melanin production [69]. Ubiquitination and deubiquitination can maintain melanogenesis by altering the catabolism or maintaining the stability of proteins associated with melanin [70]. *USP7* is involved in several protein deubiquitination bioprocesses affecting melanin synthesis. *TP53* polymorphisms affect the p53 pathway to maintain melanocyte stability. *JAK1* affects melanin production and deposition through the Jak-STAT signaling pathway [71–73]. *NF1* deficiency assays have been found to restore skin pigmentation, and thus the *NF1* gene hampers pigmentation [74]. *TET2* may affect melanogenesis by regulating 5-hydroxymethylcytosine [75]. *MYO6* and *SPIRE2* are associated with the actin filament-myosin motor system, which is linked to the transport and distribution of pigment particles [76]. MLPH proteins play an important role in the transport and distribution of melanin [77, 78]. *Rab17* delivers melanin to surrounding cells and keratinocytes by regulating the formation of filamentous pseudopods in melanocytes [79].

#### Genes associated with white wool formation

Candidate genes obtained in the white wool group focus on the differentiation of neural crest cells into melanocytes (*GLI3, REST*), proliferation of melanocytes (*CCNB1*), and melanin synthesis (*EP300, ADCY10, POU2F1, BRD4, SDHA*). *GLI3* [80] and *REST* [81] affect melanocyte formation by influencing the differentiation of neural crest cells. *CCNB1* may regulate melanocyte proliferation through the p53 signaling pathway [82, 83]. In melanocytes, *MITF* regulates *DCT* gene expression by forming a protein complex with *EP300* [84]. *ADCY10* deletion experiments demonstrated its association with the proliferation of eumelanin [85]. One study showed that *POU2F1* could control melanin synthesis by binding to *SLC7A11* to inhibit its activity [86]. Another study also showed that *POU2F1* may regulate wool color by affecting melanin production by influencing *SLC7A11, MITF, SLC24A5, MC1R*, and *ASIP* [87]. *BRD4* binds to the promoters of MITF target genes to regulate melanin production [88, 89]. *SDHA* in its homeostasis [90] may influence the state of melanin deposition in cells by regulating the mitochondrial supply of energy required for melanin synthesis.

#### Analysis of enrichment results related to wool color

Some shared regulatory pathways (Cell cycle, Cholinergic synapse, MAPK signaling pathway, ErbB signaling pathway, Oxytocin signaling pathway, Melanogenesis) were present in both wool color enrichment results, which suggests that wool color is controlled by multiple genes, and different combinations of genes will have different effects on hair color. Related terms and pathways of neurotransmitters and their receptors affect the release of pigment granules [91] such as neurotransmitter receptor metabolic process, positive regulation of dopamine receptor signaling pathway, Dopaminergic synapse [91, 92], Neuroactive ligand-receptor interaction [93], Long-term potentiation, etc. The Cholinergic synapse pathway mediates skin pigmentation and plays a regulatory role in UV irradiation-induced melanosome uptake by keratinocytes [94, 95]. The protein ubiquitination, ubiquitin protein ligase binding, and Ubiquitin mediated proteolysis affect melanogenesis by influencing the stability or activity of proteins involved in pigment synthesis. Centrosome [96] affects the transport and distribution of melanin granules in melanocytes. The microtubule and actin cytoskeleton-myosin systems co-regulate the aggregation/dispersion of intracellular pigment granules [20, 97, 98]. Dysfunction of Mitochondrion [20] is associated with hypopigmentation. Second messengers (calcium ion, cAMP, cGMP (cGMP-PKG signaling pathway)) regulate pigment aggregation/dispersion and production through protein kinase binding [20, 99, 100]. Thus, protein kinase activity, metal ion binding, kinase activity, calmodulin-dependent protein kinase activity, zinc ion binding, and negative regulation of adenylate cyclase activity all affect melanin synthesis and transport. Protein kinases influence melanin synthesis/transport, and deletion of the ATP-binding region of the protein kinase structural domain inhibits pigment dispersion; thus, ATP binding provides energy for pigment transport [100]. Methylation affects transcription factors (e.g., *MITF*) and signaling pathways (e.g., MAPK signaling pathway) associated with melanocyte migration and synthesis. lysosomal membrane is the outer membrane of the lysosome and plays a role in melanin synthesis and migration. The negative regulation of the smoothened signaling pathway involved in dorsal/ventral neural tube patterning is associated with neural crest formation and may affect melanocyte formation. Negative regulation of cell migration may affect the evenness of melanin distribution in the skin, resulting in patchy or uneven wool color. The melanocyte-stimulating hormone receptor activity and melanocortin receptor activity affect the degree of melanocyte-stimulating hormone binding, leading to an increase in melanin synthesis. The trans-Golgi network and Golgi membrane are essential for the correct processing and transportation of tyrosinase for the process of melanin synthesis [101–103]. This process ultimately leads to the formation of melanin in melanosomes, which affects the color of skin and hair. Tyrosine kinase activity is directly related to melanin synthesis, whereas serine/threonine kinase, a protein kinase C-β, is involved in the regulation of tyrosinase activity [20, 104–106]; thus, protein serine/threonine/tyrosine kinase activity influences the wool color phenotype. G-protein beta/gamma-subunit complex binding can regulate key enzymes of melanin synthesis such as tyrosinase. Arginine and proline metabolism affect and regulates the functioning of the pigment cells, which leads to the white phenotype [107]. The response to oxidative stress [108] and the HIF-1 signaling pathway [109], which is activated under hypoxic conditions, may work together to regulate melanocyte development and function, by affecting the expression of genes related to melanin synthesis. Hedgehog signaling pathway, Ras signaling pathway [110–112], MAPK signaling pathway [113–115], and Phosphatidylinositol signaling system [116] have important roles in melanocyte proliferation and differentiation. The Wnt signaling pathway is involved in melanin stem cell differentiation [117–119], melanin synthesis [114], and pigment aggregation [118]. The Notch signaling pathway affects the proliferation and apoptosis of melanoblasts [120] and melanogenic stem cells [121]. Oligopeptides such as oxytocin stimulate melanogenesis, and the Oxytocin signaling pathway regulates oxytocin production and release [20]. The Melanogenesis pathway refers to the process of melanin synthesis which is important for regulating skin and wool color [122]. The proteasome activator complex and Proteasome pathway would regulate tyrosinase processing and transport [123]. Melanocyte differentiation is closely related to the Cell cycle. ErbB signaling pathway affects pigment cell migration [124, 125].

#### Role of shared genes

Shared genes play multiple roles in wool color formation, resulting in their simultaneous presence in two candidate genes for wool color. The expression of such genes may affect the amount of pigment cell deposition and thus the shade of wool color. Mutations or variations in some genes may result in different wool color phenotypes. In addition to this, some genes are co-regulators of black and white wool color regulation, affecting the expression or function of other wool color-related genes through a complex regulatory network, suggesting that these genes have a shared effect in the two different phenotypes. Similarly, there are complex interactions between genes, and certain combinations of genes may have synergistic effects in the expression of black and white wool color, resulting in specific wool color phenotypes. The *CAMK2D* gene is involved in cellular communication during melanogenesis [126–128]. The *RPS24* knockdown assay revealed reduced pigmentation in zebrafish [129]. The *CCDC39* gene assembles the kinesin inner arm and the kinesin regulatory complex, which plays a key role in cilia movement [130], and thus it may affect melanocyte migration. *ATP8B4* plays a role in skin pigmentation changes [131, 132]. *PPP2R5E* may regulate melanogenesis/melanocyte proliferation through Dopaminergic synapse [91, 92], AMPK signaling pathway [133], and PI3K-Akt signaling pathway [134–136]. The results based on GO and KEGG enrichment analysis revealed that genes were enriched to terms and pathways related to wool color, implying that these shared genes play a crucial role in the regulation of wool color. Some candidate genes for black and white wool color have some interactions with shared genes. Key nodes in the gene interaction network (*PPP2R5E, GLIS1, CAMK2D*) may be multifunctional in the process of wool color regulation. This suggests that they may interact with each other in a complex manner and jointly participate in the establishment and maintenance of the wool color regulatory network. Through these further analyses, we gained a more comprehensive and in-depth understanding of the roles of these shared genes in black and white wool color regulation, which provides richer information to unravel the molecular mechanisms of wool color regulation.

## Conclusions

Our study pinpointed key genes like *MC1R, MLPH, SPIRE2, RAB17, SMARCA4, IRF4, CAV1, USP7, TP53, MYO6, MITF, MC2R, TET2, NF1, JAK1, GABRR1* crucial for black wool development in Gangba sheep, influencing melanocyte formation, melanin synthesis, melanin transport. Conversely, genes such as *REST, POU2F1, ADCY10, CCNB1, EP300, BRD4, GLI3*, and *SDHA* impact white wool formation by the differentiation of neural crest cells into melanocytes, proliferation of melanocytes, and melanin synthesis. Shared genes such as *PPP2R5E, CCDC39,* and *CAMK2D* play multiple regulatory roles in the black and white wool colors. This study identifies genome-wide selection signals for black and white wool in Gangba sheep, providing information for genetic improvement and selection of Tibetan sheep.

### Supplementary Information

Supplementary Material 1: Table S1. Statistics of base information before and after filtering.

Supplementary Material 2: Table S2. Reads filter information statistics.

Supplementary Material 3: Table S3. The statistical results of each sample were compared with the reference genome.

Supplementary Material 4: Table S4. Sequencing depth of each sample.

Supplementary Material 5: Table S5. Select the genes for signal screening.

Supplementary Material 6: Table S6. GO enrichment (GBB vs GBW).

Supplementary Material 7: Table S7. KEGG enrichment (GBB vs GBW).

Supplementary Material 8: Table S8. GO enrichment (GBW vs GBB).

Supplementary Material 9: Table S9. KEGG enrichment (GBW vs GBB).

Supplementary Material 10: Table S10. GO enrichment (shared genes).

Supplementary Material 11: Table S11. KEGG enrichment (shared genes).

## Data Availability

The datasets generated and analyzed in the current project (PRJNA1087586, Have been released) are deposited in the NCBI SRA repository (http://www.ncbi.nlm.nih.gov/bioproject/1087586).
